# Conductivity Prediction Method of Carbon Nanotube Resin Composites Considering the Quantum Tunnelling Effect

**DOI:** 10.3390/ma15175982

**Published:** 2022-08-30

**Authors:** Yanfeng Wang, Yongsen Yang, Huixuan Ouyang, Xiaohua Zhao

**Affiliations:** 1 Department of Civil Engineering and Architecture, Zhongyuan University of Technology, Zhengzhou 450007, China; 2 Department of Civil Engineering, Shantou University, Shantou 515063, China; 3 State Key Laboratory of Simulation and Regulation of Water Cycle in River Basin, China Institute of Water Resources and Hydropower Research, Beijing 100038, China; 4 Foshan Nanhai District Traffic Engineering Quality Supervision Center, Foshan 528251, China

**Keywords:** carbon nanotube, electrical conductivity, resin composites, tunnelling effect, effective medium model, H–S boundary model

## Abstract

Understanding and predicting the conductivity of carbon nanotube resin composites are essential for structural health detection and monitoring applications. Due to the complexity in the composition of carbon nanotube resin composites, it is of practical significance to develop a method for predicting the conductivity with a view to design and making of the composite. In this paper, the influence of carbon nanotube tunnelling on the conductivity was investigated thoroughly, where the tunnelling conductivity effect is considered as an independent conductive phase. Then, the effective medium model and the Hashin–Shtrikman (H–S) boundary model are used to predict the conductivity of carbon nanotube resin composites. The results presented in this paper show that the developed method can reduce the prediction range of the H–S boundary model and improve the prediction accuracy of the lower bound of the H–S boundary model. The results also show that the tunnelling has little effect on conductivity prediction based on the effective medium model. Based on the results, the effects of nanotube conductivity, the aspect ratio and the barrier height on the prediction of the effective conductivity are discussed to provide a guidance for the design and making of the composites.

## 1. Introduction

Fiber-reinforced resin composites have the advantages of light weight, high strength, corrosion resistance, designability and easy construction [1,2]. These materials have attracted extensive attention in the field of civil engineering detection and reinforcement [2,3,4]. Compared with traditional reinforcement methods, the use of fiber-reinforced resin composites for structural reinforcement and repair has the advantages of fast construction speed, low cost, high efficiency and low later maintenance costs. The use of fiber-reinforced resin composite plates to strengthen and repair concrete structures has been widely used in bridges, tunnels, building structures and other projects [5,6]. However, since large-scale civil engineering facilities experience erosion due to various complex environmental factors, these structures will exhibit ageing, brittle fracture, cracking and other phenomena over time [7,8]. Therefore, it is necessary to carry out health detection and performance monitoring of the structures, especially the reinforced parts of the existing structure. The use of the change characteristics of the electrical properties of fiber-reinforced resin composites to monitor the change in stress state has become a research hotspot [7,9]. Particularly in the monitoring of structural damage or concrete crack propagation, the change in electrical properties of fiber-reinforced resin composites is used for testing, which is simple, intuitive, efficient and fast [10,11].

Compared with traditional macrofibers, carbon nanotubes have the advantages of high conductivity, large specific surface area, strong corrosion resistance and smaller scale, which can realize the micromodification of resin composites [10,12,13]. Using the change in electrical properties of carbon nanotube resin composites to monitor the stress state of the structure, first, composite resins with excellent properties should be prepared. Lin Shaofeng [13] discussed the preparation of carbon nanotube resin composites and found that when the content of carbon nanotubes is 0.5~2%, the electrical conductivity of the composites shows a rapid upwards trend. When the content of carbon nanotubes exceeds 2%, the electrical conductivity of the materials increases slowly. Moisala et al. [14] studied the effects of single-wall carbon nanotubes and multiwall carbon nanotubes on the electrical properties of epoxy resin. The results show that the multiwall carbon nanotube epoxy resin composite has a lower percolation threshold than the single-walled carbon nanotube epoxy resin composite [14]. After surface acid treatment and oxidation treatment, carbon nanotubes are more easily dispersed in epoxy resin. In addition, the introduction of functional groups into oxidized carbon nanotubes can further improve the conductivity of resin composites to prepare composites with greater conductivity [15,16]. Theoretically, the conductivity of carbon nanotube resin composites comes from the number of conductivity networks formed by the overlapping of carbon nanotubes and the tunnelling effect between carbon nanotubes. To measure this effect, it is particularly important to accurately predict the conductivity of composites. To date, there have been prediction models based on micromechanics [17,18]. However, the existing prediction models have an insufficient understanding of the tunnelling effect of carbon nanotubes, and the prediction conclusions have some limitations. In our previous work, we considered the influence of the tunnelling effect on conductivity and the piezoresistive effect and found that considering the influence of the tunnelling effect can improve the prediction accuracy of the Mori Tanaka method [19]. In order to further analyze the practicability of the method, expand the application scope, and determine the influence of relevant important parameters, we conducted further research. In this paper, we continue to extend this idea to the effective medium model and the Hashin–Shtrikman (H–S) boundary model and further analyze the influence of relevant parameters on conductivity prediction results.

## 2. Conductivity Prediction Method

The conductivity of resin materials mostly ranges from 10^−16^ to 10^−12^ S/m, which is generally considered insulating. Carbon nanotubes have extremely excellent electrical properties, and their conductivity is generally from 1 to 100,000 S/m. When carbon nanotubes are added into the resin matrix, the average spacing between carbon nanotubes gradually decreases with increasing volume fraction of carbon nanotubes. When the average spacing reaches a certain value, a tunnelling current is initiated, and the carbon nanotubes and the tunnelling current begin to form a conductive network in the matrix.

**Figure 1:**
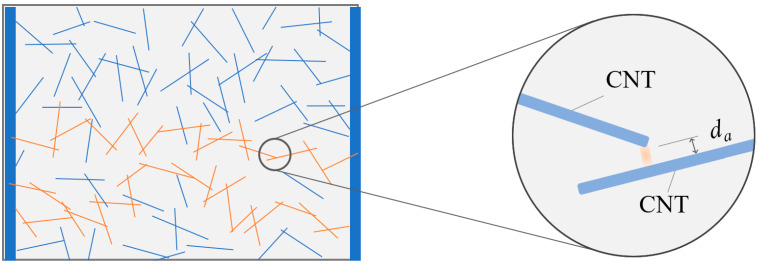
Schematic diagram of the random distribution of carbon nanotubes in resin and the average spacing between carbon nanotubes.

In a carbon nanotube resin composite, when the tunnelling effect occurs [18,19,20,21], the interaction model is shown in Figure 1 below. The average spacing between adjacent carbon nanotubes in the resin composite can be recorded as *d_a_*, which conforms to the following exponential distribution [18]:

(1) $$d_{a}=\{\begin{array}{cc} 9.8f^{-0.086}, & f<f_{cp}\\ d_{cp}\cdot {\left(f_{cp}/f\right)}^{1/3}, & f_{cp}\leq f\leq 1 \end{array}$$

In the Equation (1), f is the volume fraction of carbon nanotubes, $f_{cp}$ is the corresponding volume fraction when the percolation threshold is reached, and $d_{cp}$ is the tunnelling spacing between carbon nanotubes. In a previous study [18], a tunneling spacing of 1.8 nm is suggested. The volume fraction corresponding to the percolation threshold is related to the length-to-diameter ratio of carbon nanotubes, and the corresponding relationship is as follows [18]:

(2) $$f_{cp}=\frac{9H(1-H)}{2+15H-9H^{2}}$$

(3) $$H\left(\alpha \right)=\frac{1}{\alpha^{2}-1}\left(\frac{\alpha }{\sqrt{\alpha^{2}-1}}\ln(\alpha +\sqrt{\alpha^{2}-1})-1\right)$$

(4) $$\alpha =L/\left(2r_{c}\right)$$

where α is the aspect ratio of the CNT, L is the length of the carbon nanotube and $r_{c}$ is the inner diameter radius.

The tunnelling conductance between carbon nanotubes can be recorded as [18]:

(5) $$\sigma_{m}=\frac{e^{2}{\left(2m\gamma \right)}^{1/2}}{h^{2}}\exp(-\frac{4\pi d_{a}}{h}\left(2m\gamma {)}^{1/2}\right)$$

The specific values of parameters (*m*, e, γ, *h, d_a_*) in the above Equation (5) can be found in [18]. The conductivity of the polymer is between 10^−16^ and 10^−12^ S/m.

Therefore, in previous studies, the influence of tunnelling in the polymer is often ignored. Now, we take the tunnelling conductivity as the second phase to replace the conductivity of the polymer, the effective conductivity of the polymer can be obtained in a new way. The effective medium model and the H–S boundary model can be expressed as following [18,22]:

The effective medium model:

(6) $$\frac{\left(1-f\right)\left(1-n_{e}\right)}{n_{e}+\left(1-n_{e}\right)/3}+\frac{f}{3}\left[\frac{2\left(n-n_{e}\right)}{n_{e}+\left(n-n_{e}\right)S_{11}}+\frac{n-n_{e}}{n_{e}+\left(n-n_{e}\right)S_{33}}\right]=0$$

where $n_{e}=\sigma_{e}/\sigma_{m}$, $n=\sigma_{cnt}/\sigma_{m}$,

(7) $$S_{11}=\left\{ \begin{array}{l} \frac{\alpha }{2{\left(\alpha^{2}-1\right)}^{3/2}}\left[\alpha {\left(\alpha^{2}-1\right)}^{1/2}-\cosh^{-1}\alpha \right],\;\;\alpha >1\\ \frac{\alpha }{2{\left(1-\alpha^{2}\right)}^{3/2}}\left[\cos^{-1}\alpha -\alpha {\left(1-\alpha^{2}\right)}^{1/2}\right],\;\;\alpha <1 \end{array}\right.$$

In the above Equation (6), $S_{33}=1-2S_{11}$ and $\sigma_{cnt}$ is the conductivity of the carbon nanotube. The $\sigma_{m}$ can be obtained by Equation (5), and *α* is the aspect ratio of the CNT.

H–S boundary model:

(8) $$\sigma_{e+}=\sigma_{cnt}\left[1+\frac{\left(1-f\right)\left(\sigma_{m}-\sigma_{cnt}\right)}{f\left(\sigma_{m}-\sigma_{cnt}\right)/3+\sigma_{cnt}}\right]$$

(9) $$\sigma_{e-}=\sigma_{m}\left[1+\frac{f\left(\sigma_{cnt}-\sigma_{m}\right)}{\left(1-f\right)\left(\sigma_{cnt}-\sigma_{m}\right)/3+\sigma_{m}}\right]$$

where the parameters (f, $\sigma_{m}$, $\sigma_{cnt}$) are the same to those of the Equation (6). Equations (8) and (9) are used to calculate the H–S upper- and lower-bound model conductivity, respectively.

The solution process of the effective conductivity of carbon nanotube resin composites is as follows:

First of all, to determine the relevant parameters of carbon nanotube resin composites, such as α, $\sigma_{cnt}$, f, m, e, γ, h, $d_{cp}$ and $\sigma_{0}$.

Secondly, to calculate the percolation threshold according to Equation (2), and then calculate $d_{a}$ according to Equation (1).

Finally, $\sigma_{m}$ is calculated. If $\sigma_{m}$ is greater than $\sigma_{0}$, then substitute $\sigma_{m}$ into Equations (6), (8) and (9) to solve $\sigma_{e}$, and the resin conductivity is $\sigma_{m}$, not $\sigma_{0}$. If $\sigma_{m}$ is less than $\sigma_{0}$, let $\sigma_{m}$ = $\sigma_{0}$; then, to solve $\sigma_{e}$.

## 3. Numerical Results and Discussion

### 3.1. Validation and Comparison with Experiments

**Figure 2:**
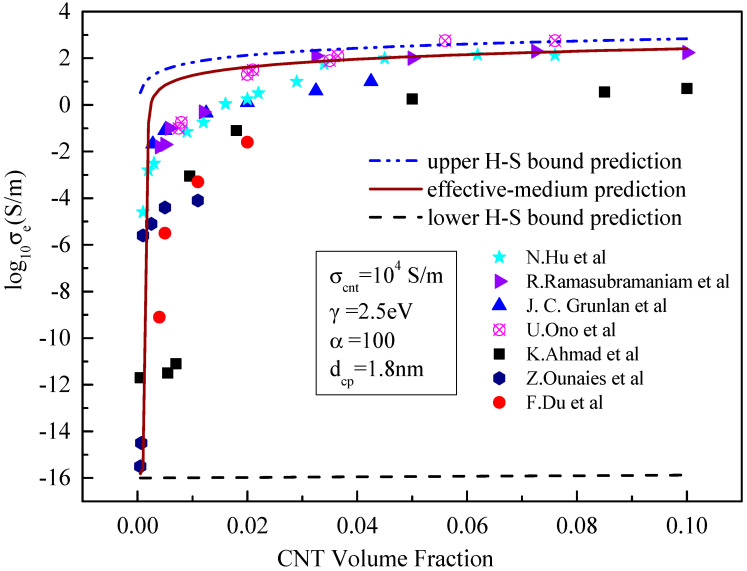
Comparison between the prediction results and existing experimental data [21,23,24,25,26,27,28] without considering the tunnelling effect.

**Figure 3:**
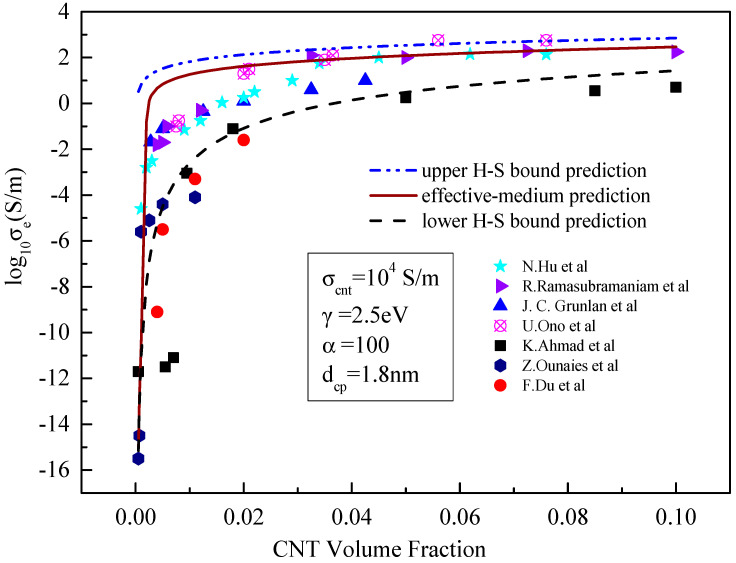
Comparison between the prediction results and existing experimental data [21,23,24,25,26,27,28] considering the tunnelling effect.

In this Section, the developed model is verified, with experimental results which are shown in Figure 2 without tunnelling effect and Figure 3 with tunnelling effect. In both figures, $\sigma_{cnt}$ = 10^4^ S/m, γ = 2.5 eV, α = 100 and $d_{cp}$= 1.8 nm. An insulating bisphenaol-F epoxy resin (jER806, Japan Epoxy Resins, Co., Ltd.) and an amine Hardener (Tomaido 245-LP) were used at a ratio of 1:2. The preparation process of the polymer can be found in [21].

It can be seen from Figure 2 and Figure 3 that the tunnelling effect of carbon nanotubes has little impact on the prediction results of the effective medium model but has significant impact on the results predicted by the boundary model, especially the lower boundary model. When the tunnelling effect of carbon nanotubes is not considered, the results predicted by the H–S lower-bound model change little with the increase in the volume of carbon nanotubes. This means that carbon nanotubes resin composites are almost insulated with the conductivity of approximate 10^−15^ S/m. When the tunnelling effect is considered, the results predicted by the H–S lower-bound model increase sharply and then gradually to more than 10^−2^ S/m with the increase in the CNT content. At the same time, the predicted results show an obvious seepage phenomenon when the content of carbon nanotubes increases. When the volume fraction of carbon nanotubes is greater than 0.01, experimental results concentrate between those predicted from lower- and upper-bound models. Since the lower bound prediction is close to experimental results, accuracy of the prediction is significantly improved.

When the tunnelling effect between carbon nanotubes is considered, the results predicted by the effective medium model are also close to the experimental results. This means that neglecting the influence of the tunnelling effect on matrix conductivity in previous studies [26,27,28] may lead to large errors in predicting the conductivity, This is consistent with our previous findings [19].

It should be noted that we use the literature data [21,23,24,25,26,27,28] to verify the validity of the calculated model. Since the temperature of this composites changes slowly during engineering application, we do not consider the influence of temperature.

### 3.2. Parametric Study

The production processes of carbon nanotubes are complex and diverse which leads to great differences in their characteristics. As such the conductivity and the aspect ratios of carbon nanotubes produced by different processes are different. When carbon nanotubes are mixed into resin materials, the performance of the composites varies significantly. To investigate the effect of different conductivity and the aspect ratios of carbon nanotubes on the performance of composites, a sensitivity analysis is carried out, given that the barrier height γ is between 1.0 and 5.0 eV according to [24]; the aspect ratios of carbon nanotubes are 50, 100, and 200, and the conductivities of the carbon nanotubes are 100, 1000 and 10,000 S/m. The results are presented here.

**Figure 4:**
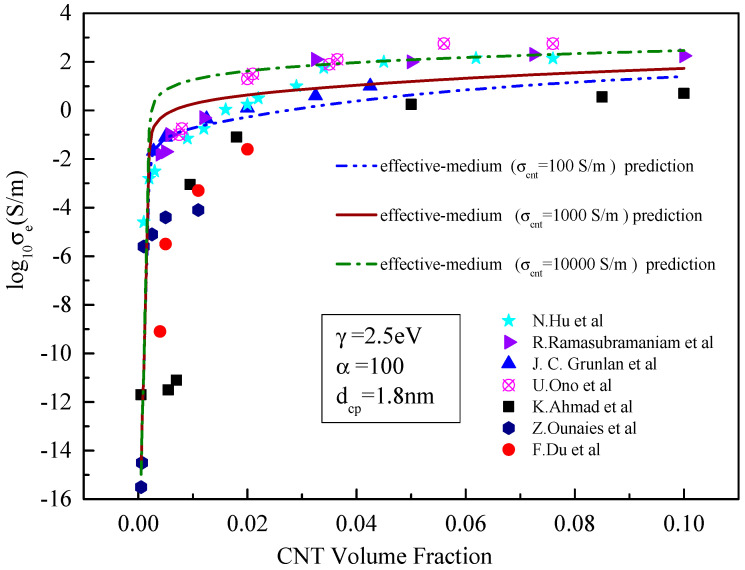
Comparison between the prediction results of the effective medium model and existing experimental data [21,23,24,25,26,27,28] with different conductivity of the CNT.

As can be seen from Figure 4, for the effective medium model, the increase in carbon nanotube conductivity has little effect on the performance of carbon nanotube resin composites before the percolation threshold but has a significant effect after exceeding the percolation threshold. When the conductivity of carbon nanotubes is 100, 1000 and 10,000 S/m, respectively, all three predicted conductivity curves contain an abrupt phase and a stable phase, with the abrupt phase basically identical to all three cases. It can also be seen that the composites with the lowest conductivity of carbon nanotubes reach the stable phase first, and the composites with the highest conductivity the last. The percolation threshold is near 0.003~0.008. At this point, the results predicted by the effective conductivity differ by approximately an order of magnitude, corresponding to the given conductivity of carbon nanotubes. This may be because after reaching the percolation threshold, the influence of the volume fraction of carbon nanotubes on its conductivity is less than that of its own conductivity, and the conductivity of carbon nanotubes plays a dominant role [27,28].

**Figure 5:**
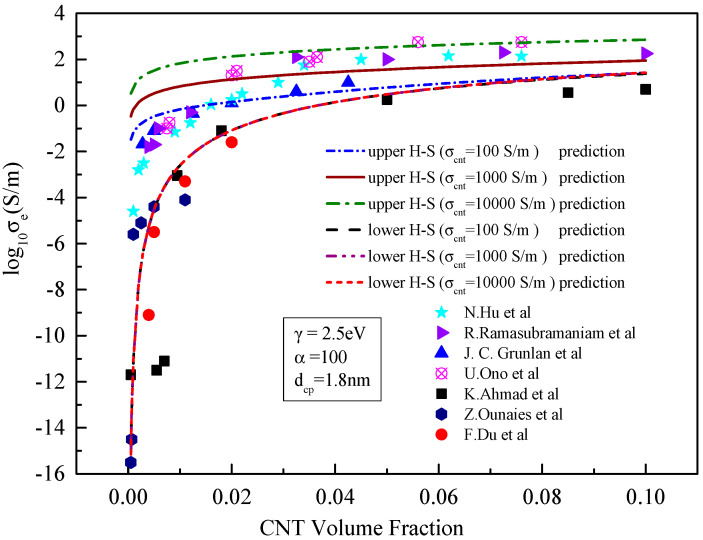
Comparison between the prediction results of the upper- and lower-bound models and existing experimental data [21,23,24,25,26,27,28] with different conductivity of the CNT.

Figure 5 shows that when the tunnelling effect is considered, the greater the conductivity of CNTs, the greater the effective conductivity of the composite, and the magnitude of increase is close. As can be seen in Figure 5, the results predicted by the H–S lower-bound model hardly change with the increase o in f the intrinsic conductivity of carbon nanotubes. When the conductivity of carbon nanotubes increases from 100 to 10,000 S/m, the value predicted by the H–S lower bound is basically unchanged, with the three predicted curves almost identical. The change in conductivity of carbon nanotubes has a great influence on the results predicted from H–S upper bound theory, with the predicted conductivity close to the intrinsic conductivity of carbon nanotubes. When the conductivity of carbon nanotubes increases by an order of magnitude, the effective conductivity of composites predicted by the H–S upper bound theory increases by an order of magnitude correspondingly. From Figure 5, when the conductivity of carbon nanotubes increases to 10,000 S/m, most of the experimental results on conductivity obtained from the literature fall within the range predicted by the upper and lower limit models. This is because that this conductivity value is adopted by most literatures [23,24,25,26,27,28].

**Figure 6:**
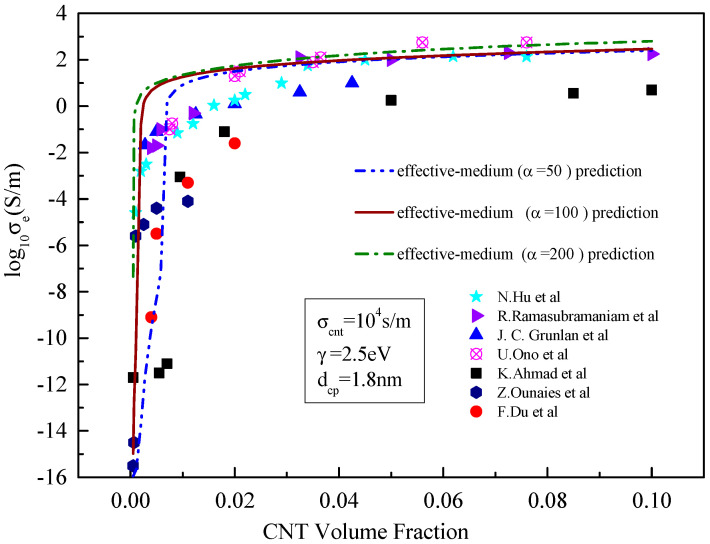
Comparison between the prediction results of the effective medium model and existing experimental data [21,23,24,25,26,27,28] with the variable CNT aspect ratio.

The influence of the length-to-diameter ratio on the conductivity of carbon nanotube resin composites is shown in Figure 6, given all other parameters unchanged. Considering the influence of the tunnelling effect, when the CNT length-to-diameter ratio is 50, the percolation threshold predicted by the conductivity of the effective medium model is approximately 0.005. When the length-to-diameter ratio of carbon nanotubes increases, the percolation threshold decreases. For the same volume fraction of carbon nanotubes, the larger the aspect ratio of carbon nanotubes, the larger the value predicted by the effective conductivity.

**Figure 7:**
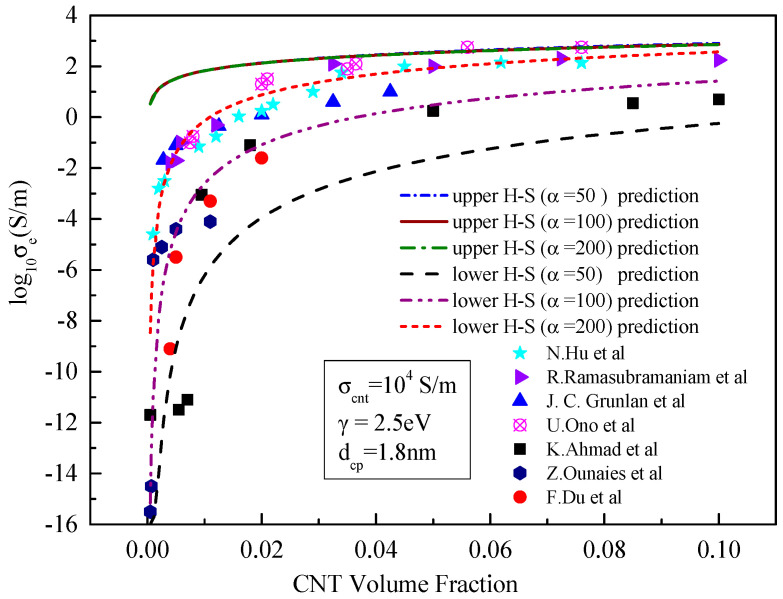
Comparison between the prediction results of the upper- and lower-bound models and existing experimental data [21,23,24,25,26,27,28] with the variable CNT aspect ratio.

From Figure 7, it can be seen that, when the volume fraction of carbon nanotubes is small, the change in its aspect ratio has a great impact on the effective conductivity of the composites. When the volume fraction exceeds a certain value, an increase in the aspect ratio leads to the gradual decrease in the predicted effective conductivity growth. From viewpoint of physics, when the conductive network in a substance is sparse, it is easier for carbon nanotubes with a larger aspect ratio to form a conductive network. Therefore, increasing the aspect ratio increases the probability of forming a conductive network, and hence the conductivity significantly. When the volume fraction of carbon nanotubes is larger than percolation, the influence of the change in the aspect ratio on the conductive network in the composite resin matrix is reduced. When the aspect ratio is increased, the increase in the effective conductivity becomes slower. Figure 7 shows that the results predicted by the upper bound of the H–S model are close to the conductivity of pure carbon nanotubes, and the change in the aspect ratio has little effect on the predicted results. On the other hand, the change in the aspect ratio has a great influence on the results predicted by the H–S lower-bound model. With the same carbon nanotube volume fraction, when the aspect ratio increases, the conductivity predicted by the H–S lower bound also increases and the percolation threshold decreases, which becomes closer to the H–S upper bound. From Figure 7, it can also be seen that, when the length-to-diameter ratio of CNTs is from 100 to 200, the predicted results by the lower-bound model are close to the test results obtained in the literature [23,24,25,26,27,28]. In this case, most of the test data are between the range of upper and lower bounds as predicted.

**Figure 8:**
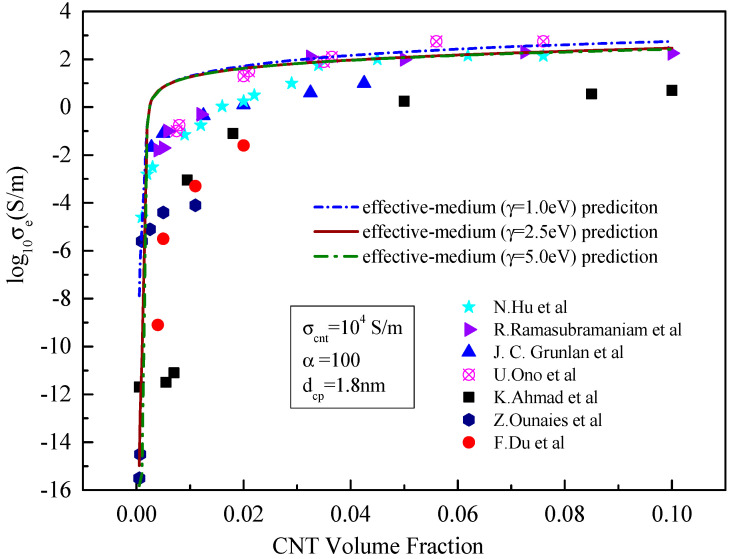
Comparison between the prediction results of the effective medium model and existing experimental data [21,23,24,25,26,27,28] with the variable barrier height.

**Figure 9:**
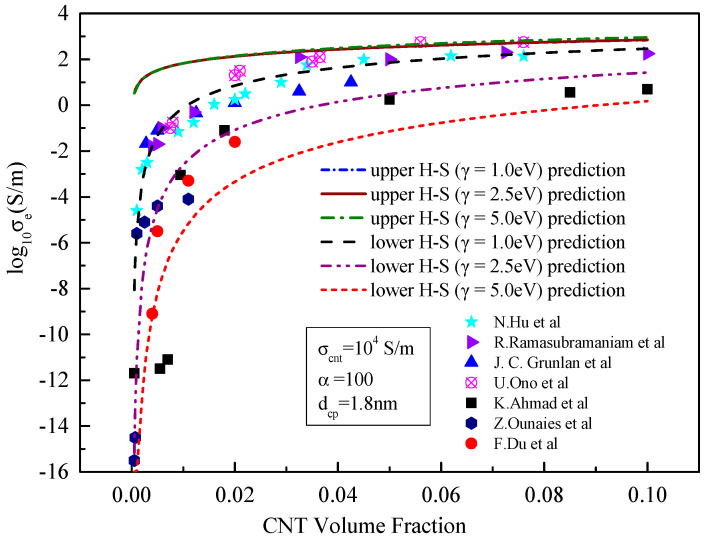
Comparison between the prediction results of the upper- and lower-bound models and existing experimental data [21,23,24,25,26,27,28] with the variable barrier height.

It can be seen from Figure 8 that different barrier heights have little effect on the prediction of the effective conductivity by the effective medium model. As the barrier height decreases, the effective conductivity in the percolation zone increases slightly, and the predicted effective conductivity also increases slightly when a certain volume fraction exceeded, but this effect is small. More specifically as shown in Figure 9, when the barrier height increases from 1.0 to 5.0 eV, the conductivity predicted by the upper bound of H–S hardly changes, and the conductivity predicted by the lower bound of H–S decreases. The possible reason for the decrease in conductivity is that the higher the barrier height, the more the energy required for electrons to move around adjacent carbon nanotubes. Therefore, the lower the barrier height, the easier the tunnelling effect is in adjacent carbon nanotubes. This is consistent with the conclusion from the literature [29]. Moreover, the lower the barrier height, the closer the predicted results are between the lower and upper bounds of the H–S model. According to Equation (8) of the upper-bound model, the conductivity of carbon nanotubes is the dominant factor affecting the upper bound conductivity. Thus, the change in matrix conductivity has little effect on conductivity of the composites. Since the effect of the barrier height on the effective conductivity is in the same order of magnitude as that of the matrix, which is much smaller than the conductivity of carbon nanotubes, it is the reason that the results predicted by upper bound seem hardly change.

## 4. Conclusions

In this paper, a novel method to predict the conductivity of carbon nanotube resin composites was developed. This method was applied to the effective medium model and the upper- and lower-bound models. The effectiveness of this method was analyzed and compared with existing experiment data. The effects of related parameters on the prediction results of the effective medium model and H–S boundary model were compared. The following conclusions are provided as follows:

(1) This method has a relatively large impact on the H–S boundary model, which improves the prediction accuracy and has a very small impact on the effective medium model. The lower bound prediction of H–S shows an obvious percolation phenomenon with increasing carbon nanotubes. When the volume fraction of carbon nanotubes is greater than 0.01, the distance between the lower bound prediction and the upper bound prediction decreases, and the prediction accuracy of the lower bound prediction is significantly improved.
(2) In general, the conductivity of carbon nanotubes, the length-to-diameter ratio and the barrier height between carbon nanotubes have important effects on the two models, especially on the upper- and lower-bound models, and on the speed of reaching the percolation threshold. Specifically, with increasing conductivity of carbon nanotubes, the predicted value of the H–S upper-bound model increases, and the order of the increase is close to that of carbon nanotubes. However, the prediction of the H–S lower-bound model and the effective medium model are not affected by the change in conductivity of carbon nanotubes. Moreover, for a given volume fraction of carbon nanotubes, the larger the length-to-diameter ratio of carbon nanotubes, the greater the predicted value of carbon nano resin composite conductivity. This change is consistent with both models, but it is especially significant for the H–S lower-bound model. Finally, when the barrier height increases, the conductivity predicted by the H–S lower-bound model decreases gradually, and the predicted value of the H–S upper-bound model changes little. Relatively speaking, the barrier height has little effect on the effective conductivity prediction of the effective medium model.

## Data Availability

Data are contained within the article.
